# Novel insights into mitochondrial gene rearrangement in thrips (Insecta: Thysanoptera) from the grass thrips, *Anaphothrips obscurus*

**DOI:** 10.1038/s41598-017-04617-5

**Published:** 2017-06-27

**Authors:** Hangrui Liu, Hu Li, Fan Song, Wenyi Gu, Jinian Feng, Wanzhi Cai, Renfu Shao

**Affiliations:** 1 Department of Entomology, China Agricultural University, Beijing, 100193 China; 2 Key Laboratory of Pest Monitoring and Green Management, Ministry of Agriculture, Beijing, 100193 China; 3 Australian Institute for Bioengineering and Nanotechnology, The University of Queensland, St Lucia, QLD 4072 Australia; 4 Key Laboratory of Plant Protection Resources and Pest Management of Ministry of Education; Entomological Museum, Northwest A&F University, Yangling, Shaanxi Province 712100 China; 5 GeneCology Research Centre, Centre for Animal Health Innovation, School of Science and Engineering, University of the Sunshine Coast, Maroochydore, Queensland 4556 Australia

## Abstract

We sequenced the mitochondrial (mt) genome of the grass thrips, *Anaphothrips obscurus*, which is highly rearranged and differs from the four thrips species reported previously in the arrangement of both tRNA genes and a protein-coding gene, *nad3*, and in the copy number of the control region (CR). We reconstructed the phylogeny of the thrips with mt genome sequences, and used it as a framework to gain insights into mt genome evolution in thrips. It is evident that *A. obscurus* is less rearranged in mt genome organization than the other four known thrips. *nad3* is in its ancestral location in *A. obscurus* but was translocated in other four thrips. Also, *A. obscurus* has one CR, which is ancestral to hexapods whereas other thrips have two or three CRs. All of the five thrips whose mt genomes have been sequenced to date are from the subfamily Thripinae, which represents about a quarter of the species richness in the order Thysanoptera. The high variation in mt genome organization observed in a subfamily challenges our knowledge about animal mt genomes. It remains to be investigated why mt genomes evolved so fast in the subfamily Thripinae and how mt genomes evolved in other lineages of thrips.

## Introduction

Most bilateral animals have the typical circular mitochondrial (mt) genome, 13–17 kb in size, containing 37 genes (i.e. 13 proteins, two rRNAs and 22 tRNAs) and a control region^1, 2^. Variation in the length of mt genomes is usually due to the length variation in the control region, which ranges from 70 bp (Orthoptera: *Ruspoliadubia*)^3^ to 4.6 kb (Diptera: *Drosophila melanogaster*) in insects^4^. Due to the small size, abundance in copy number, maternal inheritance mode, and fast evolutionary rate (up to 10 times faster than nuclear genomes^5, 6^), mt genomes have been explored widely in systematic, phylogenetic, diagnostic, and evolutionary studies^7–13^.

To date, more than 1,100 species from 28 of the 32 orders of insects (from RefSeq database) have been sequenced for complete mt genomes. Most of the insect mt genomes are highly conserved in genome organization and retain the ancestral condition for hexapods, or derive slightly from the ancestral condition^1, 14^. Major changes in mt genome organization, however, have been found in three paraneopteran orders: Pthiraptera (parasitic lice)^15, 16^, Psocoptera (booklice and barklice)^17, 18^, and Thysanoptera (thrips)^19, 20^. Fragmented mt genomes, which comprise 9–20 minichromosomes, have been found in parasitic lice^21, 22^; bipartite mt genomes have been found in booklice (*Liposcelis*)^23^ and the yellow tea thrips^20^. In both the parasitic lice and the booklice, the mt genomes are highly rearranged relative to the ancestral condition of hexapods.

The order Thysanoptera contains nearly 6,000 species^24^. A large number of these species (mostly in the family Thripidae) are phytophagous, feeding on plant tissues; some of them are crop pests or vectors of viral diseases^25, 26^. Also, many species of thrips (mostly in the family Phlaeothripidae) are fungivorous, feeding on fungi. Most of the other thrips are omnivorous, feeding on a wide range of food resources including mosses, gymnosperms, pollen, and arthropod prey^27^. Four species of thrips have been sequenced for mt genomes so far; all of them are highly rearranged and differ from each other but share the same arrangement of the protein-coding and rRNA genes except in one strain of the yellow tea thrips, *Scirtothrips dorsalis* ^19, 20, 28, 29^. The mt genomes of two strains of *S. dorsalis* have been sequenced; the typical single circular chromosome was found in the East Asia strain (EA1) while the South Asia strain (SA1) has the mt genome consisting of a large circle with 35 genes and a small circle with only three genes^20^. In addition, duplication or triplication of control region was found in the mt genomes of these thrips reported previously^19, 20, 28, 29^.

In this study, we sequenced the complete mt genome of the grass thrips, *Anaphothrips obscurus*, which is a cosmopolitan pest of cereal crops in many parts of the world^30^. Like the four other thrips species reported previously, *A. obscurus* also has a highly rearranged mt genome and furthermore, a different arrangement of protein-coding genes from the four thrips species reported previously. We reconstructed the phylogeny of *A. obscurus* with other four thrips species using mt genome sequences, and used the phylogeny as a framework to gain insights into mt genome evolution in thrips.

## Results and Discussion

### The mitochondrial genome of the grass thrips, *A. obscurus*

**Figure 1:**
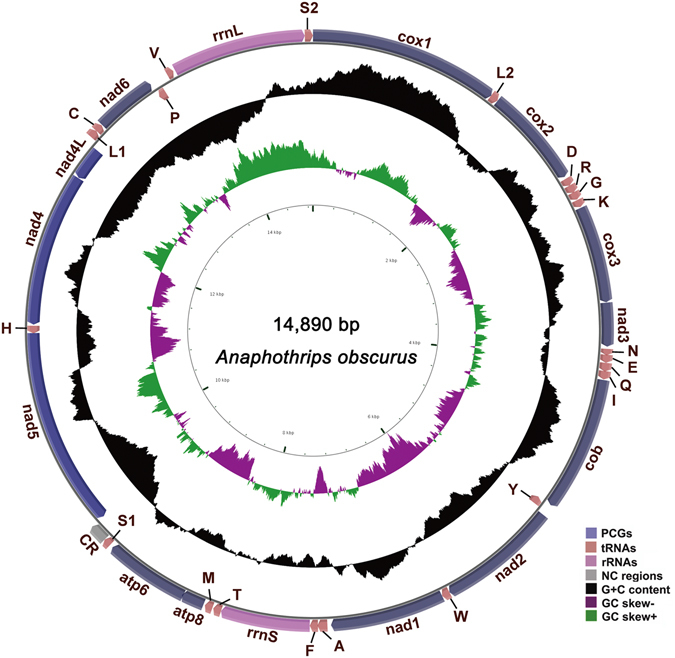
The mitochondrial genome of the grass thrips, *Anaphothrips obscurus*. Direction of gene transcription is indicated by arrows. Protein-coding genes are shown as dark blue arrows, rRNA genes as orchid arrows, tRNA genes as coral arrows and large NC regions (>100 bp) as grey rectangles. tRNAs are labeled according to their single-letter abbreviations. The GC content is plotted using a black sliding window, as the deviation from the average GC content of the entire sequence. GC-skew is plotted using a colored sliding window (green and orchid color), as the deviation from the average GC-skew of the entire sequence. In the very inner cycle, the nucleotide positions in the mitochondrial genome start from the 5′-end of *cox1*.

**Figure 2:**
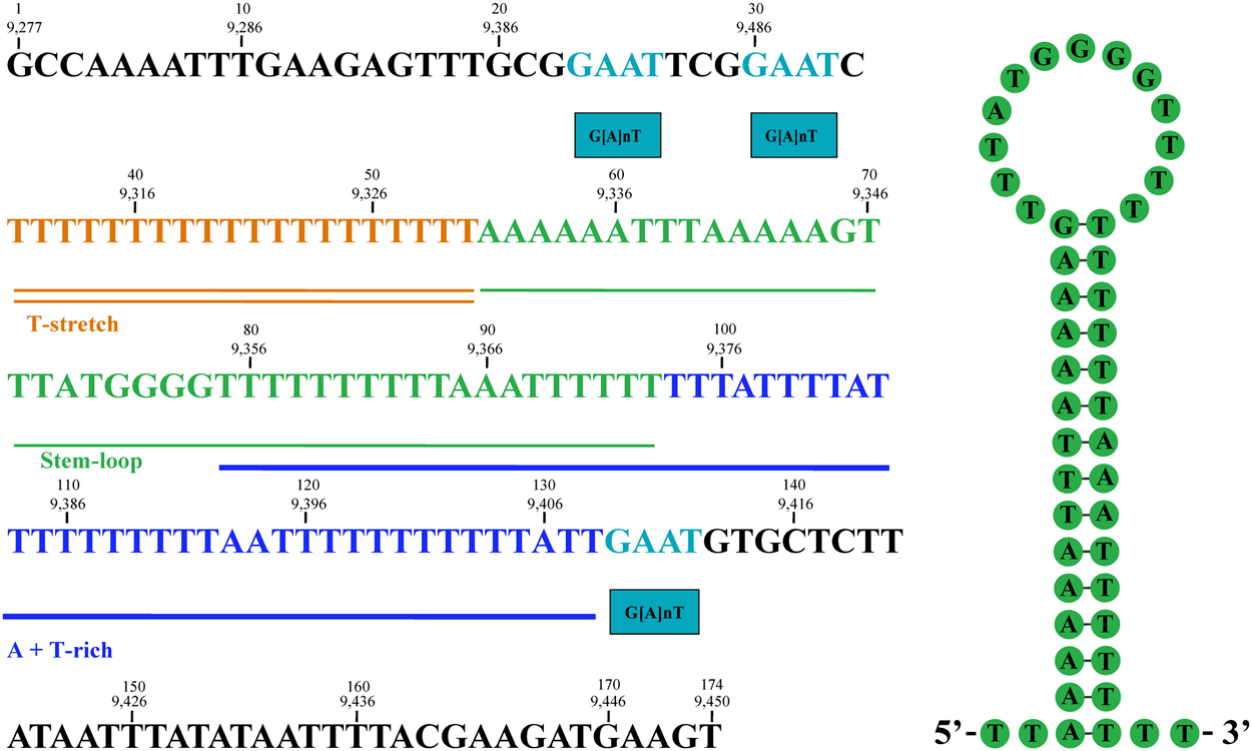
Sequence features in the mitochondrial control region of the grass thrips, *Anaphothrips obscurus*. T-stretch is shown as double line, stem-loop as thin line, AT-rich sequence as thick line and G(A)nT motifs as boxes with blue color. The secondary structure of stem-loop is shown in the right of the figure. The numbers above sequences in the upper row indicate nucleotide positions starting from the 5′-end of control region; the number in the lower row indicate nucleotide positions starting from the 5′-end of *cox1* in the mitochondrial genome.

**Figure 3:**
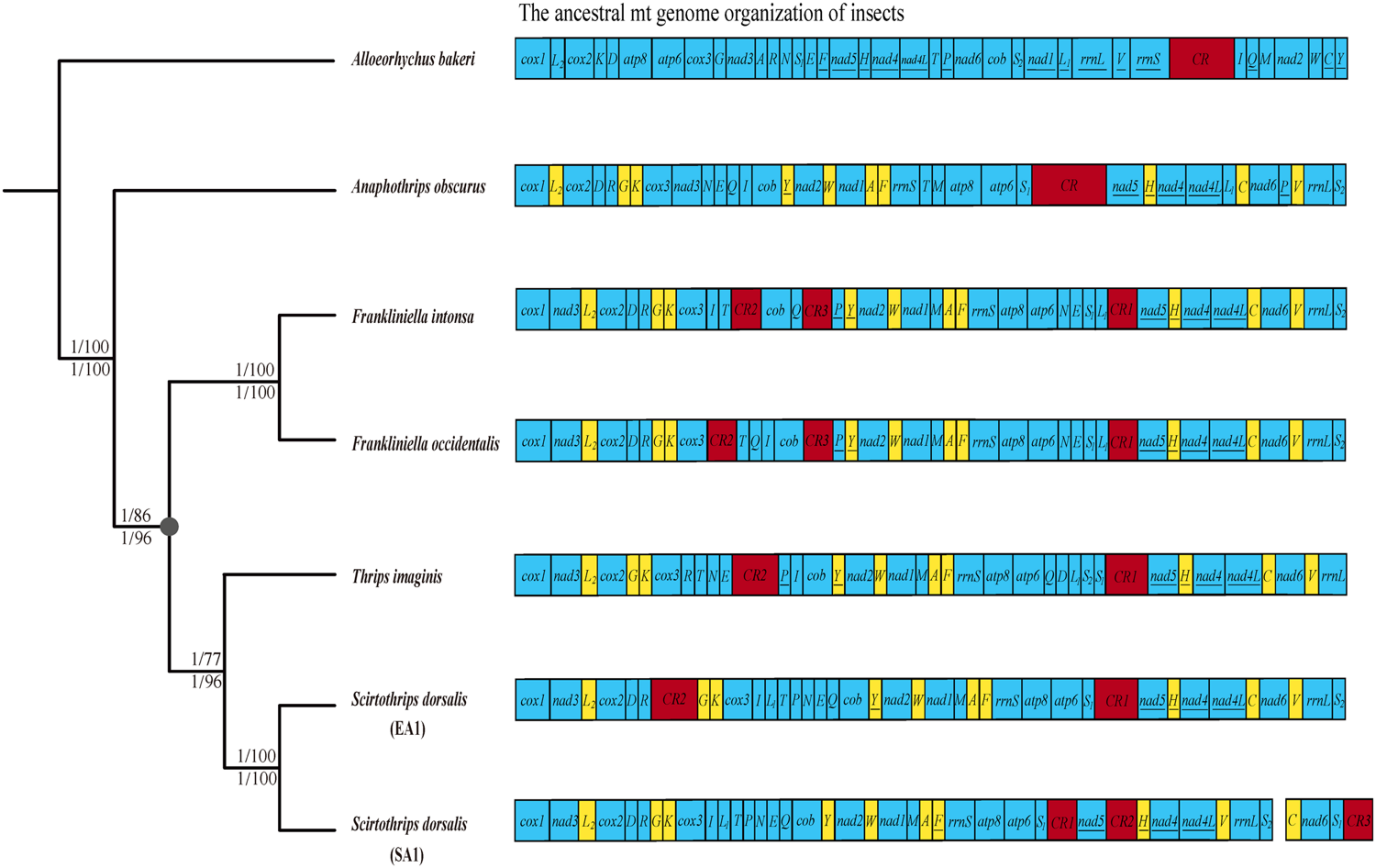
The phylogenetic tree inferred from the mitochondrial genome sequences of five species of thrips (left) and linearized mitochondrial genome organization of each thrips (right). Numbers at the nodes are ML bootstrap values and Bayesian posterior probabilities from the PCGR (upper) and PCG12R (lower) datasets. tRNA genes are abbreviated using their corresponding one-letter amino acid names. The CRs were marked as red boxes. The tRNA genes that are conserved among the five species of thrips in their location relative to their upstream and/or downstream genes were in yellow. Genes are transcribed from left to right except those underlined, which are transcribed from right to left. A grey dot at the node indicates the MRCA of the four thrips (*F. intonsa*, *F. ocidentalis*, *A. obscurus* and *S. dorsalis*) and the origin of the translocated *nad3*.

The full-length mt genome of *A. obscurus* was obtained by assembling the sequence reads from the two long PCR fragments, which were 7,188 bp and 7,409 bp in size respectively, together with the sequenced short PCR fragments, *cox1* (759 bp) and *rrnS* (450 bp). Like in most animals, the mt genome of *A. obscurus* is circular, 14,890 bp in size and contains 37 genes (for 13 proteins, two rRNAs, 22 tRNAs) and a CR (Fig. 1). The mt genome of *A. obscurus* is A and T rich with 38.4% A, 39.8% T, 10.6% C and 11.3% G. Thirty-one genes are on one strand (majority strand) and six genes are on the other strand (minority strand). Ten pairs of genes appear to overlap by 1 to 10 bp: *atp8-atp6*, *atp6-trnS*_*1*_, *cox1-trnL*_*2*_, *trnH-nad5*, *trnD-trnR*, *trnR-trnG*, *trnN-trnE*, *trnQ-trnI*, *trnA-trnF*, and *trnL*_*1*_ *-trnC*. Most of the tRNA genes can be folded into the conventional cloverleaf structure except for *trnS*_*1*_, *trnV* and *trnN*, in which the DHU arms form a loop, instead of a stem-loop. The CR is 174 bp long between *trnS*_*1*_ and *nad5*, and is highly A and T rich (83.3%). Several features commonly found in the mt CRs of insects are present in *A. obscurus*, including a stem-loop, an AT-rich segment, a T-stretch, and three G[A]nT motifs^31^ (Fig. 2). Like other four species of thrips reported previously^19, 20, 28, 29^, *A. obscurus* also has a highly rearranged mt genome. Only three ancestral mt gene clusters of insects, containing nine genes in total, are retained in *A. obscurus*: *cox1-trnL*_*2*_ *-cox2*, *atp8-atp6*, and *nad4L-nad4-trnH-nad5*; all other genes have rearranged relative to the ancestral mt genome organization of hexapods (Fig. 3).

### Phylogenetic relationships among five species of thrips inferred from mt genome sequences

To help understand the evolution of mt genome organization in thrips, we inferred the phylogenetic relationships among the five thrips species whose mt genomes have been sequenced. The maximum likelihood (ML) and Bayesian inference (BI) trees have an identical topology with strong support for each of the nodes (Fig. 3). As expected, the two species of the genus *Frankliniella*, *F. intonsa* and *F. ocidentalis*, were grouped together. The two strains of *S. dorsalis* (EA1 *and* SA1) formed a group and were most closely related to *Thrips imagini*; together, they were more closely related to the two *Frankliniella* species than to *A. obscurus*.

### Evolution of mt genome organization in thrips

**Figure 4:**
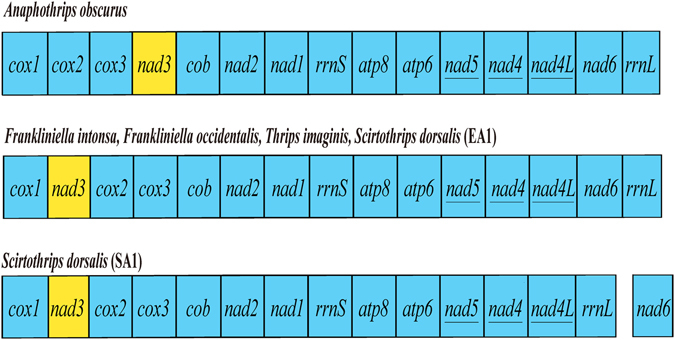
The arrangement of protein-coding and rRNA genes in the mitochondrial genomes of thrips. Genes are transcribed from left to right except those underlined, which are transcribed from right to left. tRNA genes are excluded.

Thysanoptera is one of the least studied orders among the 32 orders of insects for mt genomes. Prior to the current study, only four species of thrips have been sequenced for mt genomes^19, 20, 28, 29^. All of these thrips have highly rearranged mt genomes relative to the inferred ancestral mt genome organization of hexapods^1, 32^. Furthermore, the four species of thrips also differ from each other in mt genome organization, even between the two strains of the yellow tea thrips, *S. dorsalis* ^19, 20, 28, 29^. The mt genome of the East Asia strain (EA1) of *S. dorsalis* has a single circular chromosome whereas the South Asia strain (SA1) has two circular chromosomes: the small one is 921 bp in length and contains three genes, *nad6*, *trnC* and *trnS*_*1*_, and a CR; the larger one is 14,283 bp in length and contains all other mt genes, *trnS*_*1*_, and two CRs^20^. The variation among the EA1 strain of *S. dorsalis* and other three species of thrips is limited only to tRNA genes and CRs; the arrangement of protein-coding and rRNA genes are conserved among the four species of thrips (Fig. 4). *A. obscurus*, however, differs from the four species of thrips reported previously in the arrangement of both tRNA genes and one protein-coding gene, *nad3*. In the four species of thrips reported previously, *nad3* was translocated to between *cox1* and *trnL*_*2*_ *-cox2*. In *A. obscurus*, however, *nad3*, *cox1* and *trnL*_*2*_ *-cox2* retained their ancestral positions: *nad3* is after *cox3*; *cox1* and *trnL*_*2*_ *-cox2* are in one cluster with no genes in between (Fig. 3). As the four thrips species reported previously are more closely related to each other than either of them is to *A. obscurus*, the most plausible explanation is that the translocation of *nad3* occurred in the most recent common ancestor (MRCA) of these four thrips species (Fig. 3).

A number of studies have shown that in insects mt tRNA genes are much more mobile than protein-coding genes and rRNA genes^33–44^. Thrips are no exceptions and appear to go much further than other insects in tRNA-gene rearrangement. *A. obscurus* and the four thrips species reported previously all differ from each other in the arrangement of tRNA genes; only 10 of the 22 tRNA genes, *trnL*_*2*_, *trnG-trnK*, *trnY*, *trnW*, *trnA-trnF*, *trnH*, *trnC* and *trnV*, are conserved among the five thrips species in their locations relative to a protein-coding or rRNA gene upstream or downstream (Fig. 3). Even the two species of the same genus *Frankliniella* differ in the arrangement of three tRNA genes, *trnI*, *trnT* and *trnQ* (Fig. 3).

In addition to the genes, the CR (also called large non-coding region) also varies among the five thrips species. First, *A. obscurus* has one CR, *T. imaginis* and *S. dorsalis* EA1 have two CRs, whereas the two *Frankliniella* species and *S. dorsalis* SA1 have three CRs respectively (Fig. 3). Second, the two *Frankliniella* species and *S. dorsalis* SA1 differ in the location of one of their three CRs. *T. imaginis* and *S. dorsalis* EA1 also differ in the location of one of their two CRs. Third, the two CRs of *T. imaginis* have similar length and nearly identical sequence, so do the three CRs of each *Frankliniella* species. The two CRs of *S. dorsalis* (EA1), however, differ substantially in length (623 and 197 bp, respectively) and have no detectable sequence similarity to each other. In *S. dorsalis* SA1, the two CRs in the large mt chromosome differ in length (181 bp and 242 bp respectively) and there is no detectable sequence similarity between them. The CR3 in the small mt chromosome has high sequence similarity (98.35%) to part of CR1 on the large mt chromosome but differ in overall length (181 bp and 344 bp respectively). The CR upstream *nad5* is present in all of the five thrips species and thus can be inferred to be ancestral to these thrips. A duplication of CR likely occurred in the MRCA of *Frankliniella* species, *T. imaginis* and *S. dorsalis*, which produced CR2 between *cox3* and *cob*; the different location of CR2 in *S. dorsalis* EA1 and *S. dorsalis* SA1 can be accounted for by a translocation event of CR2. Another duplication of CR occurred likely in the MRCA of the two *Frankliniella* species, which produced CR3 between *cob* and *nad2*; in *S. dorsalis* SA1 strain, CR3 is likely due to the duplication of CR1 when the small chromosome was generated (Fig. 3).

It should be pointed out that the five thrips species whose mt genomes have been sequenced in previous studies and the current study are all from the same subfamily Thripinae of the family Thripidae. The order Thysanoptera contains nearly 6,000 described species in nine families of two suborders. The suborder Terebrantia contains eight families whereas the suborder Tubulifera contains only a single family^45–47^. Therefore, the unusual high variation in mt genome organization revealed so far from the five thrips species is essentially only from a small section of the order Thysanoptera. It remains to be investigated how mt genomes evolved in the thrips outside the subfamily Thripinae.

In summary, we sequenced the mt genome of the grass thrips, *A. obscurus*. The mt genome is highly rearranged and differs from the four thrips reported previously in the arrangement of both tRNA genes and a protein-coding gene, *nad3*, and in the copy number of CR. We reconstructed the phylogeny of the five thrips species with mt genome sequences, and used the phylogeny as a framework to gain insights into mt genome evolution and gene rearrangement in thrips. It is clear that *A. obscurus* is less rearranged in mt genome organization than the other four thrips species reported previously. *nad3* is in its ancestral location in *A. obscurus* but was translocated in other four thrips. Also, *A. obscurus* has one CR, which is ancestral to hexapods whereas other thrips species have two or three CRs. Finally, all of the five thrips species whose mt genomes have been sequenced to date are from the subfamily Thripinae, which represents about a quarter of the order Thysanoptera in terms of species richness. The high variation in mt genome organization observed so far in the subfamily Thripinae is very unusual and goes against what we know in most other animals. It remains to be investigated why mt genomes evolved so fast in the subfamily Thripinae and how mt genomes evolved in other thrips.

## Methods

### Sample collection and amplification of mt genome

Specimens of *A. obscurus* were collected at Northwest A&F University, Yangling, China. Thrips specimens were stored at −80 °C in 95% ethanol. Genomic DNA was extracted from individual thrips using DNeasy Tissue kit (QIAGEN). Fragments of mt *cox1* (759 bp) and *rrnS* (450 bp) were amplified by PCR with conserved primer pairs mtd6-mtd11 and 12SF-12SR (see Supplementary Table S1). These amplicons were sequenced at the Australian Genome Research Facility (AGRF). Two pairs of *A. obscurus* specific primers, RS45C1F-RS45rrnSF and RS45C1R-RS45rrnSR (see Supplementary Table S1), were then designed from the sequences of *cox1* and *rrnS* fragments. Long PCR with these specific primers amplified the entire mt genome of *A. obscurus* with two gaps filled by the *cox1* and *rrnS* fragments (42 bp and 251 bp respectively).

PrimeSTAR MAX DNA Polymerase (Takara) was used in the short PCR; the cycling conditions were: 94 °C for 1 min; 35 cycles of 98 °C for 10 sec, 50 °C for 15 sec, 68 °C for 30 sec, and 72 °C for 2 min. EmeraldAmp HS PCR Master (Takara) was used in the long PCR; the cycling conditions were: 94 °C for 1 min; 40 cycles of 98 °C for 10 sec, 60 °C for 30 sec, 68 °C for 8 min and 72 °C for 15 min. Negative controls were executed with each PCR experiment to detect DNA contamination and false positive amplicons. PCR amplicons were checked with 1.5% agarose-gel electrophoresis and were purified with Wizard® SV Gel and PCR Clean-Up System for sequencing.

### Next-generation sequencing, mt genome assembly and gene identification

The two long PCR amplicons, 7,409 bp and 7,188 bp in size, were obtained at different time and were sequenced separately. The 7,409-bp amplicon was sequenced with Illumina Hiseq 2000 platform at the BGI, Hong Kong. The 7,188-bp amplicon was sequenced with Illumina Hiseq 2500 platform at the Berry Genomics, Beijing. Illumina sequence-reads obtained from the long PCR amplicons were checked for quality and then assembled into contigs with Geneious 6.0.6^48^; the assembly parameters were: minimum overlap identity 98%; no gaps; maximum mismatches per read 2%; maximum ambiguity 2; and minimum overlap 100 bp.

We identified tRNA genes using tRNAscan-SE 1.2.1^49^ and ARWEN^50^. A few tRNA genes that could not be identified by these programs were found by manual inspection for predicted anti-codon sequences and secondary structure found in other thrips. Protein-coding and rRNA genes were identified by BLAST searches of GenBank^51^. The annotated mt genome sequence of *A. obscurus* has been deposited in GenBank under accession numbers KY498001.

### Phylogenetic analysis

We inferred the phylogenetic relationship of *A. obscurus* with four other species of thrips whose mt genomes have been reported (see Supplementary Table S2). The damsel bug, *Alloeorhychus bakeri* ^52^, which retained the ancestral mt genome organization of insects, was used as the outgroup. Each protein-coding gene was aligned individually by codons using MAFFT algorithm implemented in TranslatorX with L-INS-i strategy and default settings^53^. Poorly aligned sites were removed from the amino acid alignment before translating back to nucleotides using GBlocks in TranslatorX with default settings. The rRNA genes were individually aligned using MAFFT 7.0 online server with G-INS-i strategy^54^. Ambiguous positions in the rRNA gene alignments were filtered using GBlocks v0.91^55^ with default settings. Alignments of individual genes were concatenated as two datasets: 1) PCGR dataset, containing all three codon positions of 13 protein-coding genes, and two rRNA genes (11,767 bp in total); and 2) PCG12R dataset, which is the same as the PCGR dataset except the third codon positions of protein-coding genes are excluded (8,646 bp in total).

The two concatenated datasets were analyzed using maximum likelihood (ML) method implemented in RAxML-HPC2 8.1.11^56^, and Bayesian inference (BI) method implemented in MrBayes 3.2.3^57^. Multiparametric bootstrapping analysis of 1,000 replicates was performed in RAxML based on the optimal tree with the best likelihood score and the GTRGAMMA model. For MrBayes analyses, two simultaneous runs of 10 million generations were conducted for the dataset using GTR + I + G model and trees were sampled every 1,000 generations, with the first 25% discarded as burn-in. Stationarity was considered to be reached when the average standard deviation of split frequencies was below 0.01^58^.

## Electronic supplementary material

Supplementary Information
