# Supplementary material for: Novel insights into mitochondrial gene rearrangement in thrips (Insecta: Thysanoptera) from the grass thrips, Anaphothrips obscurus
Source: Sci Rep. 2017 Jun 27;7:4284. doi: 10.1038/s41598-017-04617-5 (PMC5487348; doi:10.1038/s41598-017-04617-5)

Novel insights into mitochondrial gene rearrangement in thrips (Insecta: Thysanoptera) from the grass thrips, *Anaphothrips obscurus*

Hangrui Liu[1], Hu Li[1,2], Fan Song[1], Wenyi Gu[3], Jinian Feng[4], Wanzhi Cai[1,2*], Renfu Shao[5*]

[1]Department of Entomology, China Agricultural University, Beijing, 100193, China

[2]Key Laboratory of Pest Monitoring and Green Management, Ministry of Agriculture, Beijing, 100193, China

[3]Australian Institute for Bioengineering and Nanotechnology, The University of Queensland, St Lucia, QLD 4072, Australia

[4]Key Laboratory of Plant Protection Resources and Pest Management of Ministry of Education; Entomological Museum, Northwest A&F University, Yangling, Shanxi province, 712100, China

[5]GeneCology Research Centre, Centre for Animal Health Innovation, School of Science and Engineering, University of the Sunshine Coast, Maroochydore, Queensland 4556, Australia

[*]Correspondence and requests for materials should be addressed to W. C. (caiwz@cau.edu.cn) or R. S. (rshao@usc.edu.au).

Supplementary Information: Tables S1, S2 and Figure S1.

Supplementary Table S1. The primers used in this study

Target gene	Primer name	Nucleotide sequence (5’-3’)
cox1	mtd6	GGAGGATTTGGAAATTGATTAGTTCC
	mtd11	ACTGTAAATATATGATGAGCTCA
	RS45C1F	GGGGGAGGTGATCCAACCCTTTATCAACAC
	RS45rrnSF	CCCTAAAGTTAGTTTATACTGCCATATCC
rrnS	12SA	TACTATGTTACGACTTAT
	12SB	AAACTAGGATTAGATACCC
	RS45C1R	AAGTATAGTAATGGCTCCAGCTAATACAGG
	RS45rrnSR	TAGGTTATATGTACAAATTGCCCGTCACTC

Supplementary Table S2. The mitochondrial genomes of the species used in the phylogenetic analysis

Order/Suborder	Family/Subfamily	Species	GenBank number	Reference
Thysanoptera/ Terebrantia	Thripidae/ Thripinae	*Anaphothrips obscurus*	KY498001	This study
		Frankliniella intonsa	NC_021378	28
		Frankliniella occidentalis	NC_018370	29
		Thrips imaginis	NC_004371	19
		Scirtothrips dorsalis EA1	NC_025241	20
		Scirtothrips dorsalis SA1	KM349827, KM349828	20
Hemiptera/ Heteroptera	Nabidae/ Prostemmatinae	*Alloeorhychus bakeri*	HM235722	52

Supplementary Figure S1. Inferred secondary structure of 22 tRNAs of *Anaphothrips obscurus*. The tRNAs are labeled with the abbreviations of their corresponding amino acids. Each arm and loop is illustrated as for tRNA-Val: AA-arm for amino acid acceptor arm, T-arm for TΨC arm, V-loop for variable loop, AC-arm for anticodon arm, and D-arm for dihydrouridine arm. Dashes (–) indicate Watson-Crick bonds, and dots (.) indicate bonds between other matches.

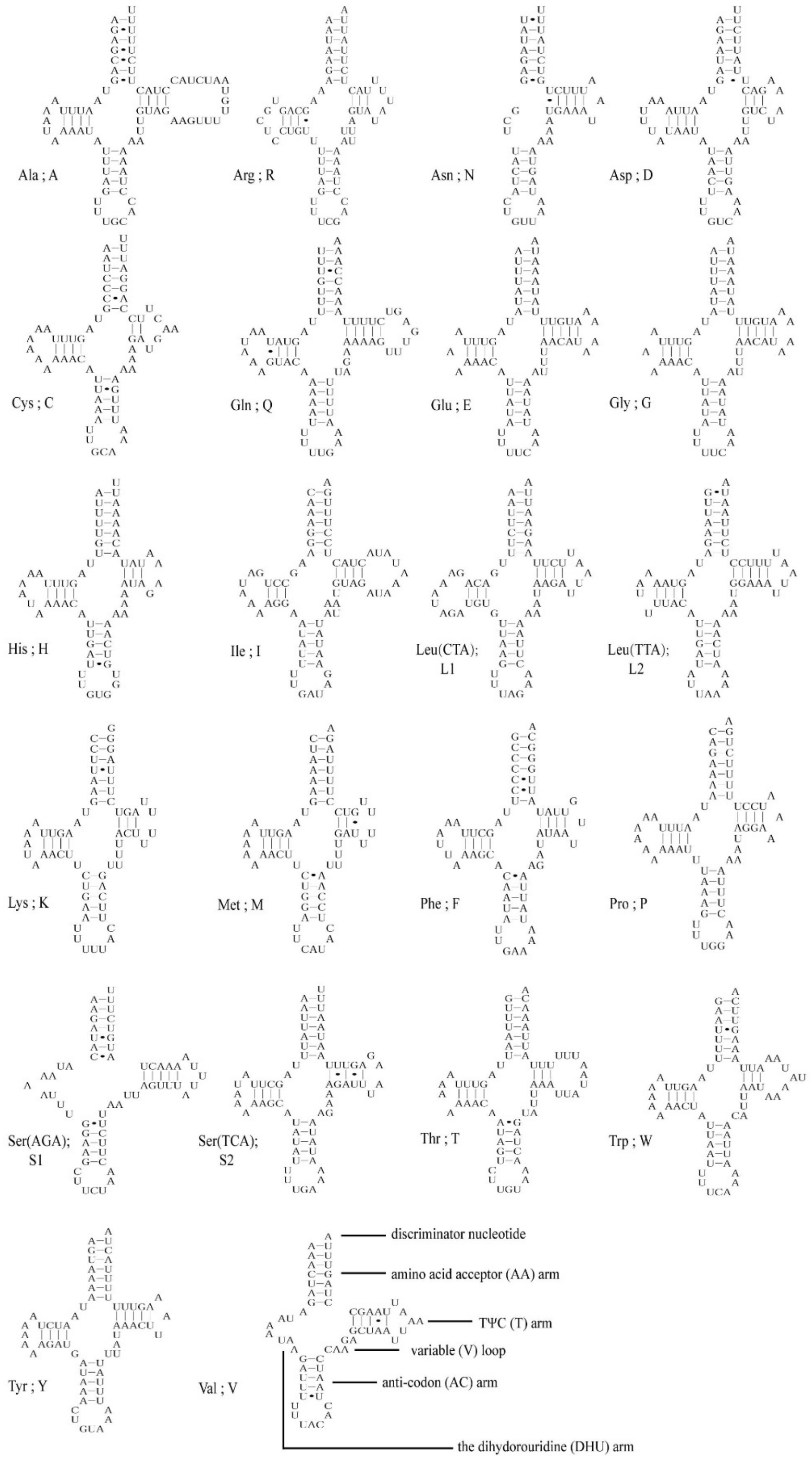

Supplement: Supplementary file 1 — Supplementary Information [file 41598_2017_4617_MOESM1_ESM.pdf]
